# Assessing willingness and preference for body scan practices in ADHD: a survey study

**DOI:** 10.1186/s12906-025-04842-w

**Published:** 2025-03-29

**Authors:** Burcu Göz Tebrizcik, Alexandra L. Georgescu, Eleanor J. Dommett

**Affiliations:** Department of Psychology, Institute of Psychiatry, Psychology and Neuroscience, King’s College London, London, UK

**Keywords:** Adults with ADHD, Meditation, Body scan practices, Treatment

## Abstract

**Background:**

Attention deficit hyperactivity disorder (ADHD) is a common neurodevelopmental condition, but current treatment options are limited. Although medication is widely used, it does not fully address all challenges and can result in considerable side effects. One approach showing promise in alleviating symptoms is mindfulness. However, typical mindfulness-based stress reduction programmes require a significant time commitment, resulting in high attrition. Researchers have therefore started to focus on discrete components on mindfulness, including the body scan meditation.

**Methods:**

Before embarking on large-scale trials, it is helpful to understand the willingness and preferences for body scan practice. Using an online survey, we examined current mindfulness exercises and preferences, willingness, belief that it was feasible to engage in body scan practices in 157 individuals with ADHD.

**Results:**

We found that most were not currently practising mindfulness but were willing to do so and believed that it was feasible. Symptom severity and available space were predictors of willingness. Preferences were shown for professional classes and 1–2 body scan practices per week. These data indicate people with ADHD are willing to engage with body scan practices, supporting the implementation of large-scale studies.

**Conclusions:**

The result provides insights to support the co-development of an intervention based on body scan meditation to manage the difficulties and the design of future studies to investigate its efficacy.

## Introduction

Attention Deficit Hyperactivity Disorder (ADHD) is characterised by inattention, hyperactivity and impulsivity [1]. It is estimated to affect 3.4% of children [2] and 2.5% of adults [3]. Consequences of ADHD include learning, behavioural and emotional problems, lower occupational status and problems with relationships [4, 5], resulting in functional impairment [6] and reduced quality of life [7]. The most effective treatment for ADHD is stimulant medication which reduces symptoms in 80% of individuals [8–10]. However, stimulants are associated with considerable side effects [11], do not completely abolish symptoms [12] and adherence can be low [13]. Whilst non-stimulant drugs exist, the response rate is lower [14], they still have side effects [11] and adherence is even poorer [15]. Given these treatment limitations, other approaches need consideration, particularly for adults, who have fewer options available [6].

One popular approach is mindfulness, a process of conscious, non-judgmental focusing of attention on the present which is thought to impact on areas of the brain associated with ADHD [16]. The standardized mindfulness-based stress reduction (MBSR) programme typically involves 26 h of classes over 8 weeks [17]. Several systematic reviews have examined the effectiveness of mindfulness in ADHD with most concluding that it may be beneficial, although more high-quality studies are needed with longer follow-up to establish the longevity of effects after formal training [18–22]. Despite the promising results of MBSR and feasible mechanisms of action, the time commitment required can make adherence challenging [23], particularly for those with ADHD who have difficulties planning [24]. Consequently, researchers have begun to focus on shorter mindfulness practices requiring less commitment.

One such practice is body scan meditation which is considered a crucial component of mindfulness [23]. A body scan involves focusing on breathing and then moving focus to different points of the body, typically head to toe [25]. Body scan practice is considered one of the most accessible components of MBSR [17] and results from several studies indicate that it increases mindfulness (see [23] for a review). Furthermore, the attrition rate is lower than for conventional MBSR [26, 27], in part thought to be due to the lower time requirements. Although some individual studies have suggested symptom improvements in clinical groups with body scan including depression [28] and anxiety [29], the balance of evidence, when subjected to meta-analyses, remains weak [23], and there is a need for high quality studies. No study has been conducted to investigate body scan efficacy in ADHD. However, before embarking on costly large-scale trials it is helpful to assess attitudes and preferences towards body scan approaches. As such, the aim of this study was to understand the acceptability of body scan meditation for ADHD. To this end we assessed existing mindfulness practices and preferences in individuals with ADHD and examined what factors predicted willingness to engage in body scan meditation approaches.

## Methods

### Participants and procedures

Data were collected using an online survey (January 2021 - July 2021). Individuals could participate if they were aged 16–35 years with a diagnosis of ADHD. The lower age limit was set to reflect a focus on adult ADHD, whilst recognising that individuals may be diagnosed using adult criteria from 17 years old, allowing a slightly lower age band [30]. The upper age limit was set to avoid age-related cognitive [31] which can impact ability to engage with mindfulness practices [32]. Participants were recruited via advertisements on volunteer sites and social media. Adverts provided a link to the study information and consent form. All participants provided informed consent before taking part in the study, including consent for the data to be published. Participants could withdraw by simply not completing the survey. Upon completion, participants could opt to enter a prize draw for a £50 voucher.

### Measures

#### Sociodemographic characteristics

Participants identified as male or female, or a term of their choosing. They reported age, ethnicity, and education level according to pre-set categories.

#### Clinical characteristics

Participants completed the 18-item Adult ADHD Self-Report Scale (ASRS) scoring statements from 0 (“never”) to 4 (“very often”) [33]. This scale has been validated in adult [33] and adolescent populations [34] making it suitable for use in our specified age group. Total (18 items, α = 0.95), inattention (IN, 9 items, α = 0.914) and hyperactivity/impulsivity (HI, 9 items, α = 0.912) scores were calculated [35, 36]. Additionally, the total for the first six so-called screener items was calculated (α = 0.863) because a score > 12 on these items is indicative of a diagnosis [33, 37]. Participants were asked to indicate if they were receiving medication and where applicable specify type (Stimulant, Non-stimulant, Other, please specify) and adherence using a previous adapted scale [38]. They were also asked if they were receiving any other form of intervention for ADHD (Y/N) with those indicating ‘Yes’ asked to provide further details.

#### Mindfulness practice

Participants indicated if they had experience of mindfulness (Y/N) and those indicating that they had were asked to specify the level (Beginner, Low, High, Expert), practice duration (< 1 month, < 6 months, < 1 year, 1–3 years, 3 years) and frequency (3–7 times per week, 1–2 times per week, once in a fortnight, once a month, less than once per month). All then completed a 13-item version of the Freiburg Mindfulness Inventory (FMI), which does not require any experience in mindfulness but assesses aspects of mindfulness through ratings from 1 (“rarely”) to 4 (“almost always”) [39, 40]. This showed excellent reliability for the total (α = 0.906) and subscale scores (presence α = 0.835; acceptance α = 0.856).

#### Body scan: willingness and preferences

Participants were provided with a definition of the body scan technique and then answered questions related to their willingness to include a 20-minute body scan as part of their condition management, and how feasible they believe it would be to regularly practice the body scan meditation. These were answered on a 5-point scale from “strongly disagree” to “strongly agree”. This was followed by a question regarding how often they would be willing to undertake body scan practice for condition management as either a standalone or adjunct treatment (3–4 times per week, 1–2 times per week, once in a fortnight, or once per month). Participants were further asked what type of body scan delivery method they preferred: Practicing independently, in a professional class, or with audio guidance from an App. Finally, information was collected about access to the required space and equipment for conducting a body scan meditation. They were asked, for instance, whether they have any quiet space in their homes or workplaces (Y/N) and their own equipment to deliver audio instructions for meditation such as headphones, computer, or mobile phone (Y/N).

### Data processing

The survey was completed by 352 individuals. Several exclusions were made before analysis. Firstly, those omitting key demographic variables (age, gender or ethnicity) were excluded (*N* = 1). Secondly, text responses that not in English were excluded (*N* = 23). Thirdly, responses with incomplete data for the ASRS (*N* = 35) or the FMI (*N* = 20) were excluded. Given that we relied on self-report we employed a cut-off score on the ASRS such that only participants scoring > 12 on the screener items were retained. Whilst this resulted in 116 exclusions, this conservative approach mitigates the risk of self-reporting a diagnosis in the absence of a formal diagnosis. The final sample size was 157. Data was analysed with SPSS.

## Results

### Demographic and clinical traits

The sample was female- and white-dominated with the most common age group 21–25 years and the most reported educational level a first degree (Table 1). The total ASRS was 52.94 ± 8.76 (M ± SD) with similar IN (26.94 ± 4.59) and HI scores (25.99 ± 5.31). All ASRS measures correlated (Total v. IN *r* =.868, *p* <.01; Total v. HI *r* =.905, *p* <.01; IN v. HI *r* =.575, *p* <.01). Seventy individuals (44.6%) were taking medication, with 48 (68.6%) taking stimulants and 20 (28.6%) taking non-stimulants. The remaining two were receiving both stimulant and non-stimulant (*N* = 1, 1.4%) or Modafinil (*N* = 1, 1.4%). Individuals taking medication had significantly higher IN (28.76 ± 4.23), *t*(155) = 4.77, *p* <.001, and total ASRS scores (55.56 ± 8.36), *t*(155) = 3.48, *p* =.001, compared to those not receiving medication (IN 25.49 ± 4.29, total 50.83 ± 8.54). There was no difference in HI score, *t*(155) = 1.73, *p* =.086. The same pattern was found for stimulants and non-stimulants, where those on stimulants had significantly higher IN (*t*(66) = 5.47, *p* <.001) and total scores (*t*(66) = 3.811, *p* <.001) but not HI scores (*t*(66) = 2.29, *p* =.054). Adherence information was provided by 53 participants. Adherence to stimulants was 75.8% ± 34.0 whilst adherence to non-stimulants was 96.7% ± 26.8, with the latter being significantly higher, *t* [50] = 2.29, *p* =.026. There was a correlation between the adherence and total ASRS (*r* = −.42, *p* <.001) with greater adherence associated with lower scores.

**Table 1:** Demographic characteristics of the final sample

	*N* (%)
Gender	
Male	66 (42.0)
Female	88 (56.1)
Other	3 (1.9)
Age (years)	
16–20	22 (14.0)
21–25	65 (41.4)
26–30	53 (33.8)
31–35	17 (10.8)
Education	
Primary education	11 (7.0)
Secondary education	23 (14.6)
Further education	36 (22.9)
Post-compulsory trade/technical/vocational	21 (13.4)
Bachelor’s degree	43 (27.4)
Master’s degree	17 (10.8)
Doctorate degree	3 (1.9)
No response	3 (1.9)
Ethnicity*	
White	101 (64.3)
Mixed	25 (15.9)
Asian	9 (5.8)
Black	20 (12.7)
Other	2 (1.3)

*Collapsed across 17 categories. No participants selected prefer not to say for gender or ethnicity

Sixty-eight participants (43.3%) reported receiving non-medication interventions with a range of approaches identified (e.g., Expressive Arts Therapy, Eye Movement Desensitization and Family Therapy). The most cited approach was CBT (*N* = 32, 47.1%). There were no significant differences in any ASRS scores between those receiving non-medication interventions and those not (IN *t*(154) = 0.99, *p* =.325; HI *t*(154) = 1.76, *p* =.08; total ASRS *t*(154) = 1.58, *p* =.116). There was a significant association between the use of medication and non-medication interventions (*χ*^*2*^ [1] = 5.91, *p* =.015) with individuals receiving medication more likely to engage in other interventions.

### Mindfulness practice

Table 2 provides an overview of current mindfulness practices. The largest single group of respondents had no prior experience with mindfulness and therefore did not currently practice it. The next largest group classed themselves as beginners. In terms of duration of practice, excluding those with no experience, the most cited response was practising for less than one month, with 1–2 per week the most common frequency. FMI data revealed an overall score of 33.34 ± 8.47 with similar presence (15.90 ± 4.17) and acceptance (17.43 ± 4.95) scores. These FMI measures all correlated (Total v. Presence *r* =.915, *p* <.01; Total v. Acceptance *r* =.940, *p* <.01; Presence v. Acceptance *r* =.723, *p* <.01).

**Table 2:** Current mindfulness practice

	*N* (%)
Experience level	
None	66 (42.0)
Beginner	47 (29.9)
Low level of experience	33 (21.0)
High level of experience	10 (6.4)
Expert	1 (0.6)
Duration of Practice	
Never	66 (42.0)
< 1 month	44 (28.0)
< 6 months	20 (12.7)
< 1 year	7 (4.5)
1–3 years	13 (8.3)
3 years +	7 (4.5)
Frequency of Practice	
Never	66 (42.0)
< once per month	14 (8.9)
Once per month	10 (6.4)
Once per fortnight	10 (6.4)
1–2 per week	33 (21.0)
3–7 per week	24 (15.3)

### Body scan attitudes

Participants reported high levels of willingness to engage in body scan techniques with 81.5% strongly or somewhat agreeing, in contrast to only 5.7% strongly disagreeing or somewhat disagreeing. There were also positive responses regarding how feasible they believed it would be with 76.7% strongly or somewhat agreeing that it would be feasible, in contrast to just 7.7% strongly disagreeing or disagreeing. One-sample Wilcoxon tests indicated a significant positive response to these items (Willingness Median = 4, *Z(*157) = 9.57, *p* <.001; Feasibility Belief Median = 4, *Z*(156) = 8.89, *p* <.001). Perhaps unsurprisingly, there were significant positive correlations between experience level and feasibility (*r* =.148, *p* =.002) and willingness (*r* =.161, *p* <.001). Preferences regarding the frequency of mindfulness practice were similar irrespective of whether it was considered as a standalone (SA) or adjunct (ADJ) treatment. In both cases the most preferred frequency was 1–2 times per week (SA *N* = 53, 33.8%; ADJ *N* = 46, 29.3%) and the least preferred was 3–4 times per week (SA *N* = 27, 17.2%; ADJ *N* = 21, 13.4%). The intermediate frequencies of once per month (SA *N* = 39, 24.8%; ADJ *N* = 42, 26.8%) and once a fortnight (SA *N* = 33, 21.0%; ADJ *N* = 44, 28.0%) were reversed in preference between the two treatment modes (SA vs. ADJ). For support methods, a professional class was the most favoured method (*N* = 60, 38.2%) followed by independent practice (*N* = 48, 30.6%) and finally use of an App (*N* = 45, 28.7%). Most had access to quiet space (*N* = 127, 80.9%) and suitable equipment (*N* = 141, 89.8%).

To establish which factors might underpin willingness to engage with body scan practice hierarchical linear regression was used. Demographic variables were entered in Step 1 (Age, Gender, Ethnicity and Education). Given the low number in some categories, only male and female gender was used, and ethnicity was considered in terms of white versus non-white. Clinical information was added at Step 2 (total ASRS, medication and non-medication treatment), with FMI score, mindfulness experience, availability of space and equipment added in the final step (Table 3). Total rather than subscale scores were used due to the high correlation between subscales. Step 1 did not account for a significant amount of the variance in body scan willingness, *R*^*2*^ = 0.045, *F*(4, 143) = 1.69, *p* =.156. However, the addition of clinical characteristics in Step 2 did explain a significant amount of the variance, *R*^*2*^ = 0.207, *F*(7, 140) = 5.23, *p* <.001, with total ASRS a significant positive predictor. The final model (Step 3) explained 26.5% of the variance in willingness, *F*(11, 147) = 4.45, *p* <.001, with ASRS score and space availability as significant positive predictors.

**Table 3:** Regression modelling of willingness to engage in body scan practice

Predictor	Δ*F* (*p*)	Δ*R*^2^	b	β	*t* (*p*)
Step 1	1.69 (.156)	.04			
Age		5	.158	.146	1.56 (.122)
Gender			−.079	−.042	−.511 (.610)
Ethnicity			.263	.145	1.58 (.117)
Education			.002	.003	.033 (.974)
Step 2	9.55 (<.001)	.16			
Age		2	.200	.185	2.12 (.036)
Gender			−.145	−.077	-1.01 (.314)
Ethnicity			.270	.138	1.74 (.085)
Education			.007	.012	.14 (.893)
Medication			.040	.022	.267 (.790)
Non-medication			.271	.144	1.736 (.085)
Total ASRS			.038	.354	4.48 (<.001)
Step 3	2.64 (.036)	.05			
Age		7	.171	.159	1.82 (.071)
Gender			−.186	−.099	−.129 (.201)
Ethnicity			.179	.092	1.14 (.257)
Education			−.009	−.015	−.17 (.866)
Medication			.047	.025	.30 (.762)
Non-medication			.252	.134	1.66 (.100)
Total ASRS Space			.037	.352	4.38 (<.001)
Space			.597	.252	2.89 (.008)
Equipment			−.311	−.104	-1.13 (.261)
Prior experience			−.090	−.094	-1.20 (.231)
Total FMI			−.014	−.129	-1.51 (.134)

## Discussion

The present study aimed to investigate current mindfulness practices and preferences for body scan approaches in individuals with ADHD to better understand how this might be received if it were offered as part of ADHD treatment and to inform future trials. Our findings demonstrated that most participants had little mindfulness experience, with those who were practising, most likely to have done so for one month or less. Perhaps unsurprisingly their mindfulness scores were not overly high (34 out of a maximum of 52) aligning with previous research which has shown mindfulness scores positive relate to prior experience [41]. However, most reported high levels of willingness to engage with body scan techniques and believed it feasible. This feasibility correlated with prior mindfulness experience. Most also had access to a quiet space and equipment to undertake body scan practice. In terms of practice preference, the most preferred frequency was 1–2 times per week, although the strength of this preference was slightly reduced for adjunct treatment. For delivery method, most preferred to receive support via in-person professional classes. Although we did not ask participants to explain their choice, this may relate to distractibility within ADHD making it hard to practice independently.

We found that ADHD severity predicted willingness to engage with body scan approaches. Whilst there is no prior research examining willingness to engage in mindfulness or body scan treatments in ADHD, prior research does indicate that condition severity is related to willingness to engage in other non-pharmacological treatments [42]. This is logical, given that those with more severe ADHD are more likely to have remaining symptoms, even with medication. The other significant positive predictor of willingness was the availability of space for body scan practice. This suggests that physical constraints should be considered in any future trials, even though most individuals here did have suitable space. We tentatively speculate that the high level of willingness to engage in body scan meditation could indicate that adherence to this type of meditation may also be high and therefore could overcome the attrition of longer MBSR programmes, which is supported by other work indicating body scan practice can be popular and have high adherence in other populations [43]. However, it is important to note that willingness may not directly translate to adherence. Indeed, studies examining meditation in other populations have found various factors impact on adherence including frequency of practice, meditative focus [44], mood and sleep [45], and personality traits [46] and so future studies should directly measure adherence using subjective and objective measures [47]. This is particularly important in individuals with ADHD who can struggle with adhering to medication [48] and homework components of psychological interventions [49]. It is also important to note that medication adherence in the present study, where reported, particularly to non-stimulants was high [38] and so the sample may not be fully representative of the wider ADHD population in terms of adherence and by inference, willingness. It is also noteworthy that our final model only explained 26.5% of the variance in willingness, suggesting that even if willingness translates to adherence, there are other important factors at play, and these should be further investigated. Perhaps unsurprisingly, despite prior experience correlating with willingness, we did not find it to be a significant predictor of willingness. It is possible that this is because most of our sample were relatively inexperienced and therefore the data range was small. As such, arguably the most important findings from the current study are the preferences for body scan practice which could inform trial designs, that is to have professional support, and practice requirements of no more than 1–2 per week for 20 min.

Although this is the first study to assess willingness and preferences for body scan in ADHD, there are limitations to the work. First, the study results may not generalise to all ages because our age range was 16–35 years old. This range was used to avoid age-related cognitive decline [50, 51]. Whilst not directly required in this study, this is part of a broader scheme of work where this was necessary. Second, the study did not consider co-occurring conditions, particularly autism spectrum disorder, depression and anxiety [52], which are commonly found with ADHD and may impact on willingness and preferences relating to body scan. Third, the study relied on self-reported diagnosis. Although there was little incentive to be dishonest and the reported ASRS scores are appropriate, we cannot be certain this did not occur. The use of the ASRS screener threshold, which has high concurrent validity with the commonly used clinician-rated ADHD Rating Scale [53], was intended to mitigate this in part, but future studies should consider confirming the diagnosis as part of the study procedure. Fourth, the recruitment allowed self-selection, and this may have introduced bias, and contributed to the gender bias as females typically engage in online research more than males [42]. Fifth, we only looked at a limited number of variables, and other factors may be more influential, such as the specific type of mindfulness practices individuals had previously engaged with. Related to this, participants were not asked to explain preferences and qualitative work could allow a more detailed exploration. Finally, the study asked participants to share their beliefs about the feasibility of using body scan practices and their willingness to do so rather than actually practising this meditation. Although this is an important first step, this research should now be built upon with feasibility and acceptability trials and larger randomised controlled trials as appropriate.

## Conclusions

Given the need for alternative interventions in ADHD, the potential of mindfulness, and concerns over MBSR time commitments, this study investigated willingness and preference for body scan meditation in ADHD. The findings suggest individuals are willing to participant in regular body scan practice. Reported preferences suggest that interventions should consider professional in-person support and practice required no more than 1–2 per week for 20 min. Based on these findings, further research assessing the feasibility, adherence and efficacy of body scan meditation in ADHD is warranted.

## Data Availability

The datasets used and/or analysed during the current study are available from the corresponding author on reasonable request.

## Abbreviations

ADHD: Attention Deficit Hyperactivity Disorder
MBSR: Mindfulness-based Stress Reduction
ASRS: Adult ADHD Self-Report Scale
IN: Inattention
HI: Hyperactivity/impulsivity
FMI: Freiburg Mindfulness Inventory
SA: Standalone treatment
ADJ: Adjunct treatment
CBT: Cognitive behavioural therapy
